# Comprehensive representation of health-related phenotypes in one million dogs using topic modelling of electronic health records

**DOI:** 10.1186/s40537-026-01365-0

**Published:** 2026-02-24

**Authors:** Peter-John Mäntylä Noble, Sean Oliver Farrell, Noura Al-Moubayed, Alan David Radford

**Affiliations:** 1 Department of Small Animal Clinical Science, Institute of Veterinary and Ecological Sciences, University of Liverpool, Leahurst Campus, Chester High Road, Neston, CH64 7TE Wirral UK; 2 Department of Computer Science, Durham University, Upper Mountjoy Campus, Stockton Road, Durham, DH1 3LE UK; 3 Department of Infection Biology and Microbiomes, Institute of Veterinary and Ecological Sciences, University of Liverpool, Leahurst Campus, Chester High Road, Neston, CH64 7TE Wirral UK; 4 School of Medicine, Stanford University, Stanford, California, USA

**Keywords:** Veterinary clinical records, Topic modelling, Phenotype discovery

## Abstract

Historically, veterinary studies screening for breed, age and sex predisposition to disease have relied on collating small-scale studies of clinical datasets. The availability of larger datasets through groups such as the Small Animal Veterinary Surveillance Network (SAVSNET) promise access to information regarding a wide range of clinical presentations at scale, however, methodological limitations surrounding the extraction of specific disease information or screening for disease predispositions result in a substantial reduction in the number of animals studied. These studies often address very focused hypotheses - only leveraging a small fraction of the intrinsic value of the data at any one time. Here, we implemented an unsupervised machine learning methodology, creating a representation of a large volume of clinical notes collected by SAVSNET from veterinary practices across the UK. We utilise BERTopic, a topic-modelling tool based on Bidirectional Encoder Representations using Transformers (BERT) architecture, and show it is able to surface known phenotypes, such as breed predispositions to hypoadrenocorticism, diabetes mellitus and mitral valve disease, as well as potential novel patterns of disease phenotypes. This scalable and granular modelling technique facilitates the rapid interrogation of large clinical datasets, enabling the identification of a broad range of phenotypes within the population and the early detection of temporal changes indicative of emerging infectious or environmental diseases.

**Supplementary Information:**

The online version contains supplementary material available at 10.1186/s40537-026-01365-0.

## Introduction

In the veterinary health literature, the identification of which breeds (ages or sexes) are affected by a specific disease or which range of diseases affect a specific breed has typically been studied using comparatively small cohorts of animals with a specific condition, for example, animal predisposition to mammary cancer [44] or of breeds of animals where records could be screened for wide-ranging diseases, for instance, diseases affecting French Bulldogs or Boxers [29, 31]. Such studies typically include hundreds to small numbers of thousands of individual animals. Similarly, modelling the occurrence of disease with age is not easily achieved at scale for many breeds at one time. Ultimately, consensus about disease in dogs is usually reached through a review of multiple publications, often incorporating comparatively small groups of animals.

Recently, extremely large datasets have become available through groups such as Vet Compass [28] and the Small Animal Veterinary Surveillance Network (SAVSNET, [38]). These hold significant volumes of canine electronic health records, which in turn will embody key patterns of phenotypes displayed by dogs as represented by the clinical narratives contained therein. While these systems allow for some disease-coding by attending clinicians, they suffer from either simple single-choice coding (SAVSNET) or complex coding choices that are associated with poor compliance, and for all such coding systems, the reliability of the attending clinician to enter this data is very variable [14, 25].

The veterinary informatics sector struggles to fund work on a scale that would allow wide-ranging manual record annotation, and some form of automation of record annotation at scale would alleviate this challenge. A potential candidate for this is machine learning (ML), which has been used to create automated record classification systems for both text and image data. However to date, these are usually supervised methods requiring both prior knowledge of the phenotypes likely to be seen and reasonably large datasets of expert-annotated records in order to train these systems [10, 26], all of which require substantive investment of time on a case by case basis. Additionally, the process by which ML systems generate classifications can often lack explainability regarding the factors that influenced the classification. This can lead to systematic errors due to the model identifying unexpected features in the data necessitating the development of systems such as LIME and SHAP to generate explanations for ML outputs [4, 36]. An ideal system would involve unsupervised annotation of records according to disease phenotypes based on intrinsic characteristics of the notes. The aim would be that explainable results would be intrinsic to the method.

We have previously shown that latent Dirichlet allocation (LDA) topic modelling allowed the identification of a specific gastroenteric disease in clinical notes from dogs that would have allowed early detection of a disease outbreak [27]. LDA is based on a bag-of-words statistical method that does not differentiate word meaning according to context, potentially limiting the ability of the technique to differentiate topics. A more modern approach incorporates a combination of neural language modelling using bidirectional encoder representations using transformers (BERT) [8] to create document embeddings with subsequent dimensionality reduction using UMAP and clustering into documents with common word-composition based topics using hdbscan. This combination of techniques is implemented in the BERTopic package [12]. In addition to creating topic models, BERTopic is able to represent topic evolution across time (dynamic topic modelling) or other classifications (e.g. breed) inherent to the data under study.

Whilst novel in veterinary studies, topic modelling has been evaluated for information extraction from human electronic health records [37] and social-care notes with clinical content [41] highlighting a clear opportunity to leverage its use for veterinary data.

In the current study we evaluated the hypothesis that topic modelling with BERTopic using records from a million dogs will allow us to identify population-wide disease phenotypes which can be analysed on the basis of a number of factors including breed, age, sex and time-course.

We address the key problem that while we have access to large volumes of clinical records, identifying key disease phenotype signals in these data presents a significant bottleneck. Our objective was to evaluate how, using a minimal approach to BERTopic customisation, a topic model based on health data from one million dogs could surface disease phenotypes in an explainable and analysable form.

We use BERTopic to create topic models based on a large subset of SAVSNET clinical narratives. We evaluate the patterns of phenotypes revealed within BERTopic-generated topics, comparing how topics for disease change with time and in specific breed, age, and sex cohorts compared to known disease occurrence and how the models perform in revealing new clinical insights.

## Materials and methods

We followed the STROBE and RECORD reporting guidelines [2, 43]. To enhance transparency regarding the ML methodology, we also incorporated relevant principles from reporting guidelines for artificial intelligence in health research [17].

The study design comprised collating a set of clinical narratives from veterinary consultations involving dogs. These were passed to a topic-modelling package (BERTopic) [12] for annotation with topics intrinsic to the clinical text. Topic coherence was assessed and subsequently a series of analyses of odds ratios for the association of given topics with breed, age, sex and time were assessed and presented graphically.

Endpoints of the study were to illustrate trends in the data corroborated primarily by 95% confidence intervals for topic associations differing from reference populations (with breeds this was usually cross breed)

### SAVSNET datasets

SAVSNET collects data from approximately 580 veterinary premises across the UK. After each patient consultation, data are passed to SAVSNET and comprise the species, breed, age, and sex of the patients, along with the clinical narrative recorded by the attending veterinarian. The current study used a random sample of one million clinical records, each record coming from a unique dog and comprising the deidentified clinical narrative, the breed, age, sex and the date of consultation.

### Topic models

Narratives from the one million record datasets were used to train a BERTopic (version 0.13.0) model [12]. Briefly, BERTopic embeds documents using the sentence transformer all-MiniLM-L6-v2 [16] followed by reduction of dimensionality using UMAP(umap-learn package v0.5.2) [24] and subsequent clustering using HDBScan(hdbscan package v0.8.37), an efficient clustering algorithm with good noise rejection and minimal parameter configuration [23]. Clusters are then analysed using term frequency/inverse document frequency (TF/IDF) to identify word weighting for each topic [33]. A trial and error method was used to adjust the UMAP and HDBSCAN parameters to generate a usable model with minimal generation of duplicated and vacuous topics (A1, A2). The topics were assessed by a clinically active academic (Noble) to attribute clinical relevance. The quality of the model was assessed using the gensim package (v4.2.0) CoherenceModel class with the ’c_v’ coherence metric [35], providing a measure of semantic interpretability. The distribution of topics by age, breed class, or consultation month was created using the topics_per_class method.

Models were trained using a Ryzen-9 12 core CPU PC with 64Gb of DDR-4 RAM equipped with an Nvidia A4000 GPU (16Gb internal memory).

### Data analysis

Using this method, each clinical narrative was assigned a probability distribution for containing any topic. For subsequent analysis, each consultation was classified according to the most probable topic for that consultation.

### Odds ratio calculation and confidence intervals

Odds ratios were calculated to quantify the association between topic presence and class membership relative to a specified reference class. For each topic-class combination, a $2 \times 2$ contingency table was constructed where *a* represents the frequency of the topic in the class of interest, *b* represents the frequency of the topic in the reference class, *c* represents the frequency of all other topics in the class of interest, and *d* represents the frequency of all other topics in the reference class. To address zero-count cells, a continuity correction of $+1$ was applied to all cell counts. Odds ratios were calculated using python code incorporated in plot generation using the following calculations:

1 $$\begin{aligned} \text {OR} = \frac{a/b}{c/d} = \frac{a \cdot d}{b \cdot c} \end{aligned}$$

Ninety-five percent confidence intervals for the odds ratios were derived using the logarithmic transformation method. The standard error of the log odds ratio was calculated as:

2 $$\begin{aligned} \text {SE}(\log \text {OR}) = \sqrt{\frac{1}{a} + \frac{1}{b} + \frac{1}{c} + \frac{1}{d}} \end{aligned}$$

The confidence intervals were then computed as:

3 $$\begin{aligned} \text {CI} = \exp \left( \log (\text {OR}) \pm 1.96 \times \text {SE}(\log \text {OR})\right) \end{aligned}$$

where 1.96 represents the critical value of the standard normal distribution for a 95% confidence level.

Topic word-contents were reviewed using the BERTopic visualize_barchart method. Plotly [39] was used to present a filterable line plot of proportions of narratives labeled with given topics broken down by age and date. For time series, values were normalised to peak values for each individual series to allow comparison. For breed and sex-related data, odds ratios along with 95% confidence intervals were calculated for the proportions of topic-labelled narratives against suitable references and plotted as tree plots using Plotly.

## Results

The SAVSNET database contained 5,467,034 narratives from dog consultations. With the hardware in use, BERTopic would overrun memory limits in the GPU when used to train the model with more than 1,000,000 records. When the number of possible topics was unconstrained, BERTopic generated a model with over 900 topics, many of which were vacuous (sequences of words with no clear clinical correlate) or very similar in word composition to other topics (e.g. multiple combinations describing a vaccination event). As a consequence, BERTopic was run, limited to producing a maximum of 200 topics which appeared to represent diverse clinical presentations with less duplication (HDBScan and BERTopic parameters are shown in supplementary material tables A1, A2).

The coherence analysis of the BERTopic model demonstrated exceptional performance, with an average c_v coherence score of 0.8501 across 197 evaluated topics. Of the 200 topics initially generated, 184 topics (93.4%) achieved good coherence scores ($\ge$0.7), while only 11 topics (5.6%) fell into the acceptable range (0.5–0.7.5.7), and merely 2 topics (1.0%) showed poor coherence (<0.5). Notably, 10 topics achieved perfect coherence scores of 1.0, indicating optimal semantic consistency within these thematic clusters. Individual topic assignment probabilities were relatively low (median maximum probability: 0.049, mean: 0.129), with only 6.9% of documents assigned with>0.5 probability to their designated topic.

The top 15 most common topic representations (15 words or word-pairs for each topic) are shown in supplementary material (Figure. SA1). Despite the count reduction, 53 topics still contained words relating to vaccination or booster.

### Breed distribution

**Fig. 1:**
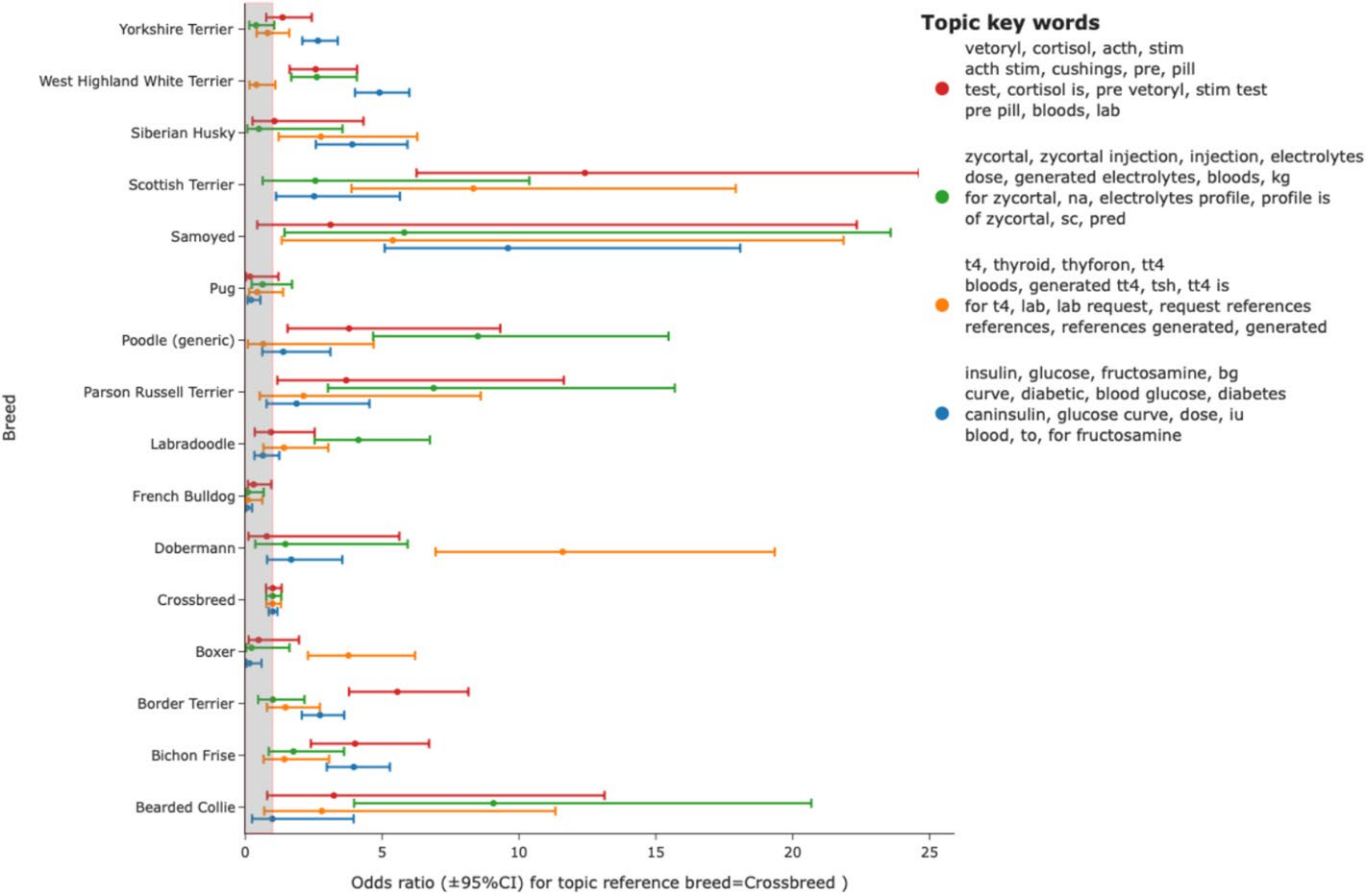
Breed distribution of endocrine disease topics. Topics representing animals probably affected by diabetes (blue), hyperadrenocorticism (red), hypoadrenocorticism (green) and hypothyroidism (orange) were readily identified by topic wording. Odds ratio (reference=crossbreed) and 95% confidence interval were plotted for a selection of breeds with potential predispositions to individual endocrinopathies

**Fig. 2:**
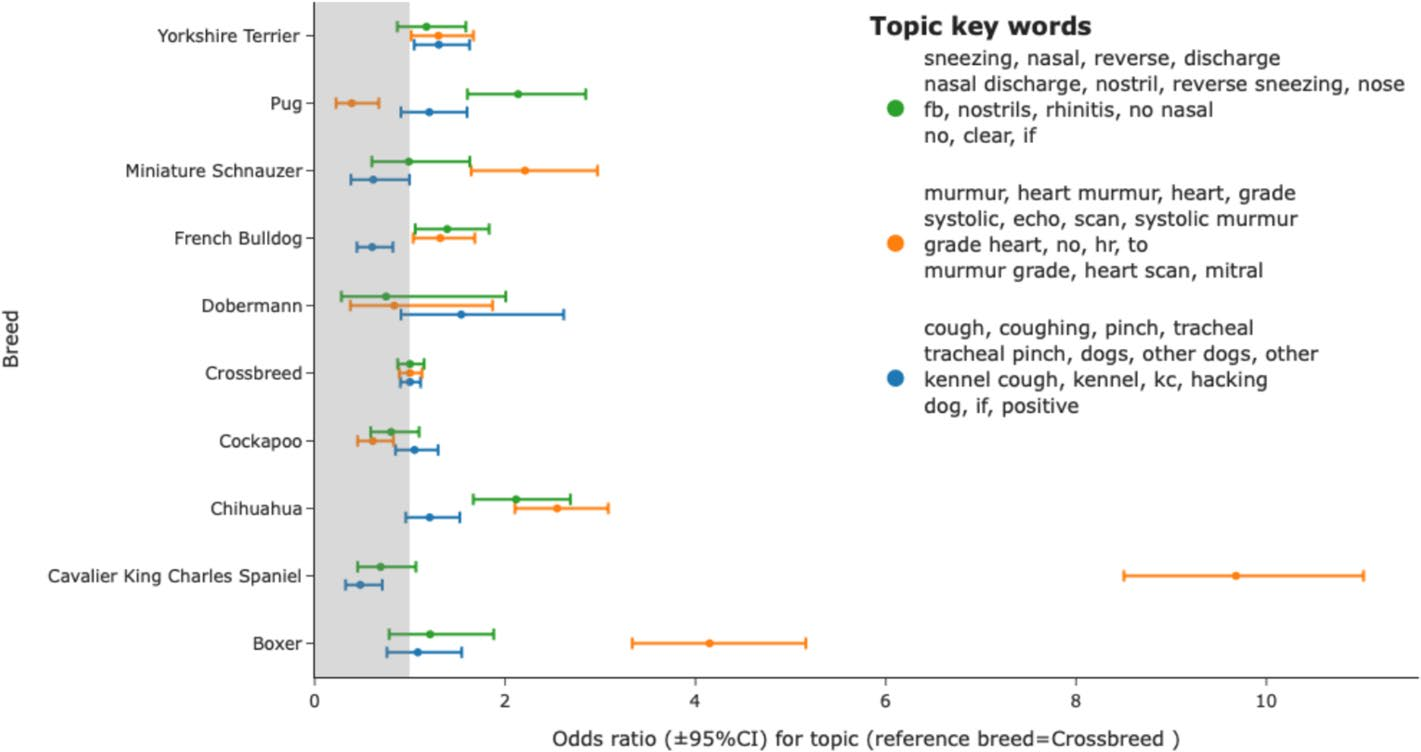
Breed distribution of cough (blue), sneeze (green) and murmur (orange) topics. Here, the distribution for topics reflecting respiratory or cardiac clinical signs are shown for a selection of breed. Odds ratio (reference=crossbreed) and 95% confidence intervals for breed having consultations matched by the given topic

SAVSNET data comprised information about 217 unique breeds. Using the topics_per_class method of BERTopic allowed the distribution of topics for any breed to be identified and odds ratios for any breed having a given phenotype calculated with crossbreed as a reference. A wide range of potential breed predispositions could be identified. An example of four topics representing different endocrine diseases is shown in Fig. 1. Here increased occurrence of diabetes topic is seen in Samoyeds, Huskies and West Highland White Terriers whereas the description of thyroid disease is seen more commonly in standard poodles, dobermans and Scottish terriers. Hypoadrenocorticism-related narratives were seen in the standard poodle, Bearded Collies, Labradoodles and German short-haired pointers (Fig. 1). Similarly, breed associations for clinical syndromes involving the cardio-respiratory system could be evaluated with an example dataset shown in Fig. 2. Example topics representing upper respiratory signs (sneezing, reverse sneezing), cardiac findings (heart murmur) and cough are shown and illustrate potential predispositions (e.g. murmur in Cavalier King Charles spaniel and Boxer and Chihuahua, sneeze in Pug and Chihuahua) and reduced instances (e.g. murmur in Pugs, cough in French Bulldog).

### Age distribution

**Fig. 3:**
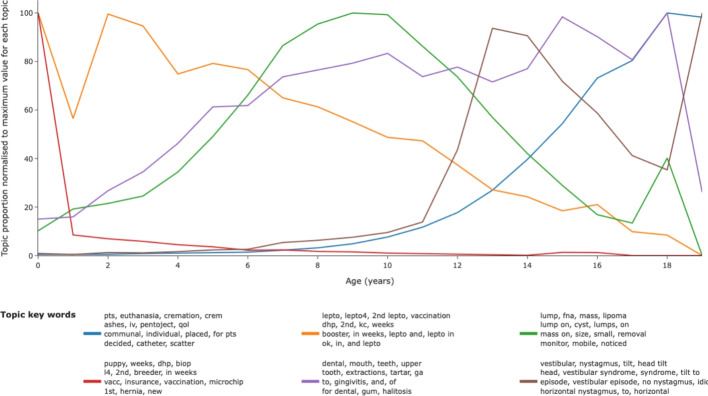
Examples of topics with distinctive age distributions. Age distribution for a group of topics that represent early-life (vaccination and puppy check), mid-life (lumps and masses) and late-life (vestibular disease and euthanasia) presentations. Each topic is normalised to its maximum proportion to allow comparison of age-pattern for each topic

SAVSNET data comprised records from animals aged 0–18 years old. Topics illustrated a wide range of age-related variation such that topics relating to vaccination and puppy health checks occurred in younger animals. Topics relating to masses occurred more commonly in middle aged dogs and topics relating to vestibular disease, and euthanasia occurred in older dogs (Fig. 3).

### Sex distribution

**Fig. 4:**
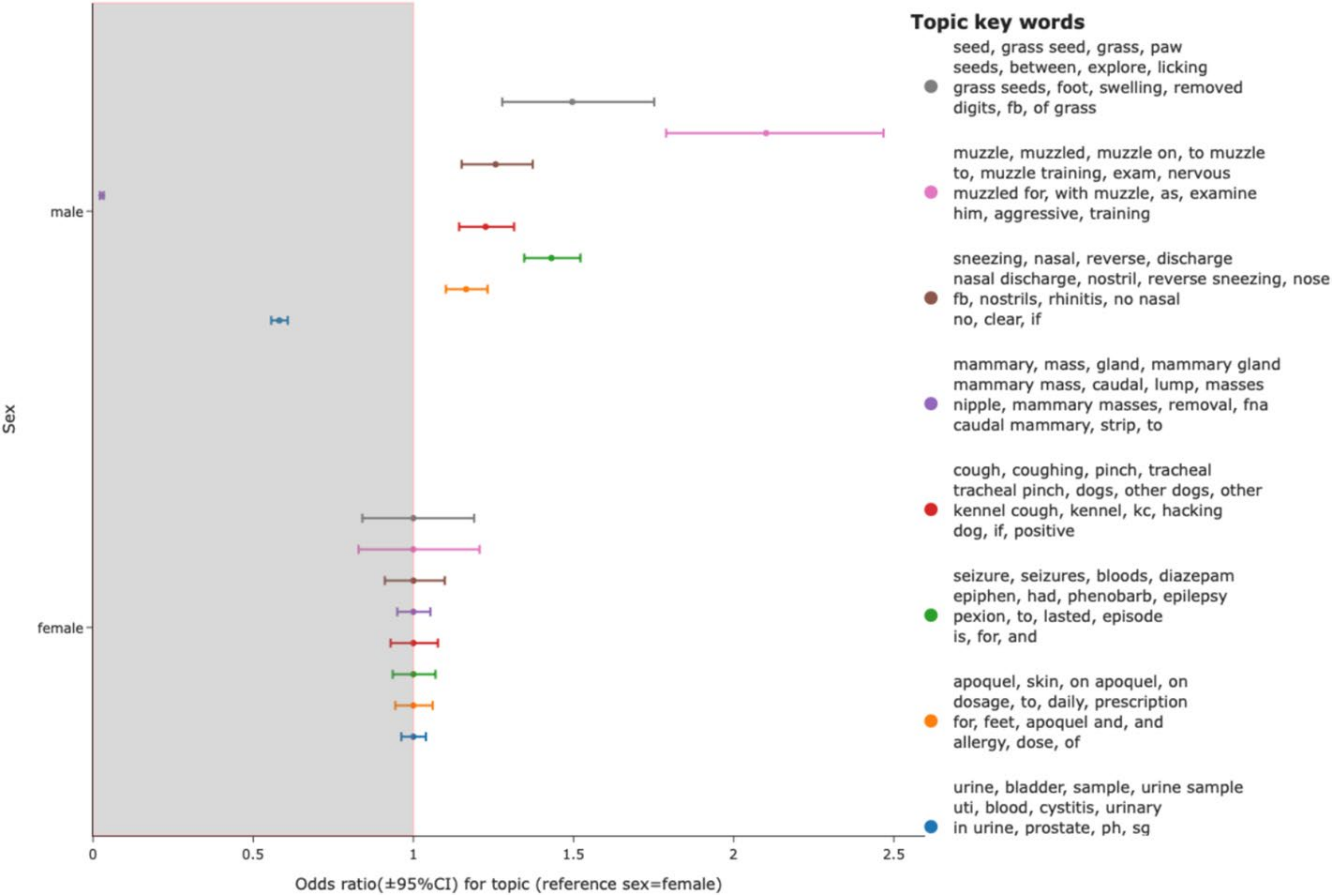
Topic distribution for male and female dogs. Selected topics are shown where odds ratios (reference=female) suggest differences between male and female dogs

Differences in topic distributions between male and female dogs were evaluated and demonstrated some variation. Odds ratios for male and female neutering were strongly (and appropriately) segregated for the relevant sexes such that the only topic containing the word spay (‘spay’, ‘season’, ‘pre’, ‘for spay’, ‘pre spay’, ‘spay check’, ‘op’, ‘pre op’, ‘last season’, ’lap’, ‘spey’, ‘vulva’, ‘check’, ‘mammary’, ‘lap spay’) had an odds ratio of 0.01 in males compared to females and in a similar way, a topic associated with castration (‘testicle’, ‘castration’, ‘castrate’, ‘testicles’, ‘pre’, ‘implant’, ‘suprelorin’, ‘both testicles’, ‘op’, ‘pre op’, ‘for castration’, ‘descended’, ‘check’, ‘for castrate’, ‘scrotum’) had an odds ratio of 13.7 in males compared to females. Additionally, odds ratios for muzzling (a likely marker of aggression), seizures, coughing, sneezing and skin disease topics were higher for males while odds ratio for urinary tract infection and mammary disease were lower in males (Fig. 4).

### Outbreak patterns

**Fig. 5:**
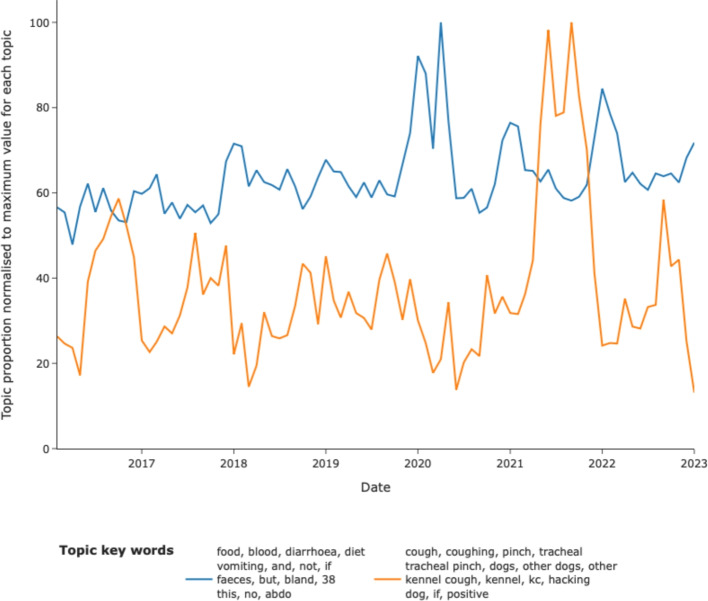
Examples of topics highlighting potential disease outbreaks. The distribution of topics with time are shown for a topic most-likely representing gastroenteric disease and one representing respiratory disease. The former reflects a national outbreak of gastroenteric disease in spring 2020, the latter, a potential national increase in respiratory disease occurring in autumn 2021

Analysis of topic occurrence with time allowed for the detection of increases in clinical syndromes on a national level (using this model). Here, a topic relating to gastrointestinal signs revealed a seasonal increase in these signs in winter with a pronounced spike in activity of this topic in winter 2020. A marked increase in activity of a topic relating to respiratory signs was seen in Autumn 2021 (Fig. 5).

### Syndrome seasonality

**Fig. 6:**
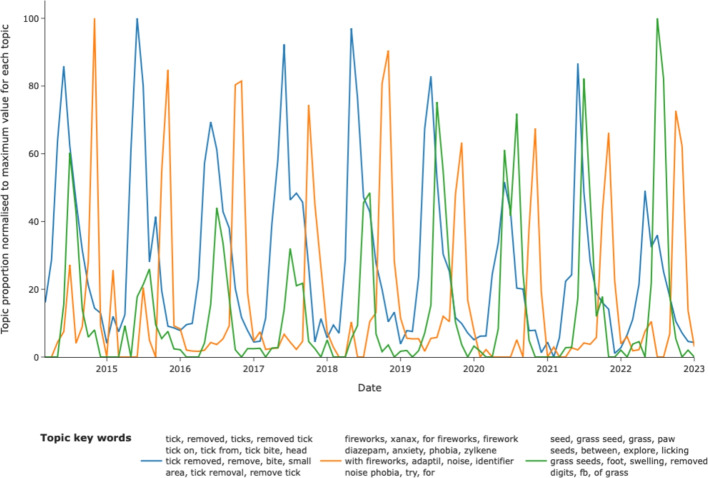
Examples of topics that illustrate detection of seasonal patterns, in this case, firework anxiety (orange), tick infestation (blue) and grass-seed trauma (green)

Given the ease of screening the patterns of topics with time, it was straightforward to identify clinical syndromes with marked seasonality. Thus topics relating to grass seed foreign bodies, firework anxiety and removal of ticks from the patient showed marked, repeatable seasonality (Fig. 6).

### Temporal trends

**Fig. 7:**
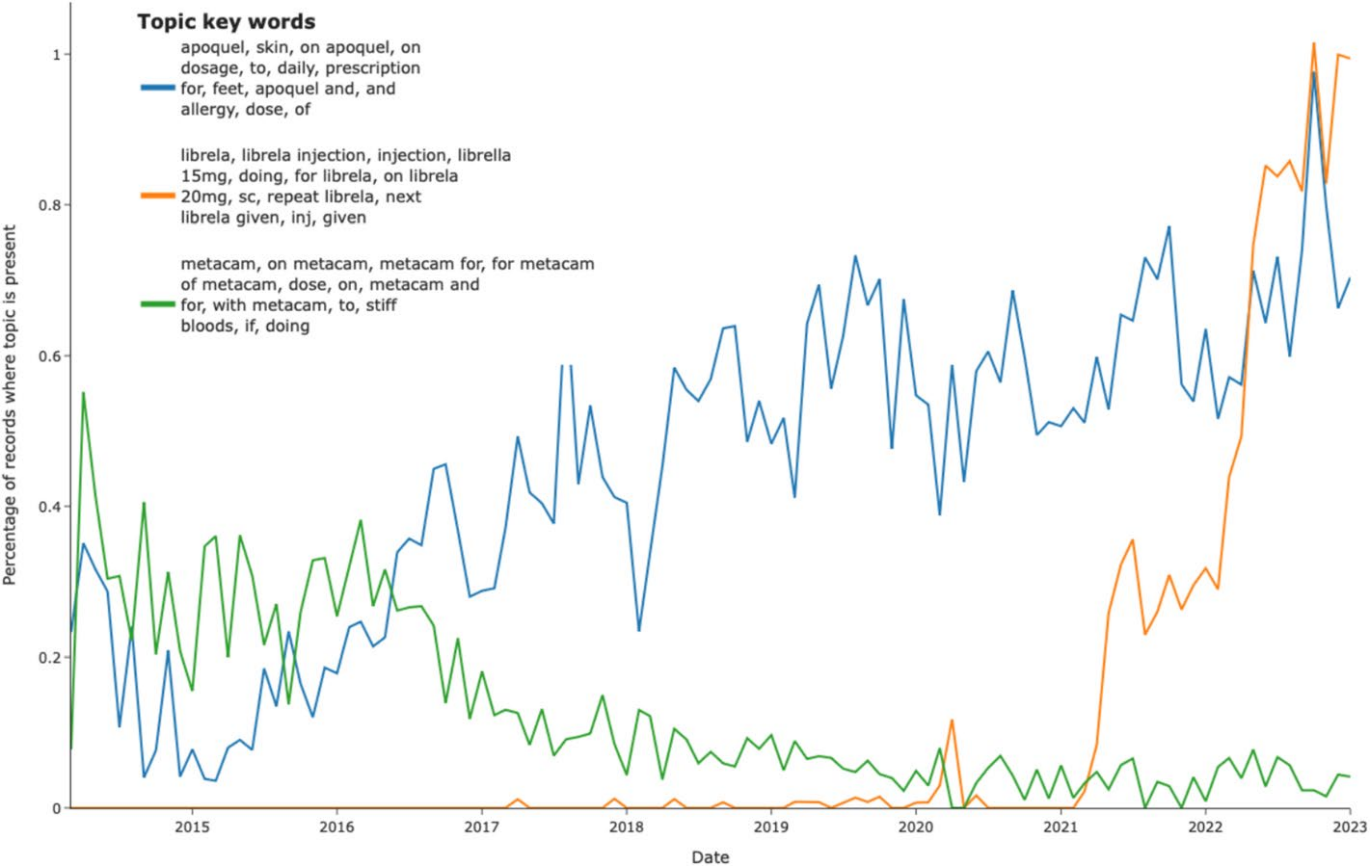
Examples of topic that show long-term trends with time. In this case consultations discussing use of drugs Occlacitinib (Apoquel®), meloxicam and bedinvetmab (Librela®)

The key words for each topic often indicated that use of a specific drug was a core feature in that topic, allowing an evaluation of trends in use of those drugs with time. Example of these trends are seen in Fig. 7 where consultations describing meloxicam usage decrease in proportion with time where the proportion of consultations describing use of occlacitinib increased steadily with time and bedinvetmab steeply after 2020.

### Effects of lockdown

**Fig. 8:**
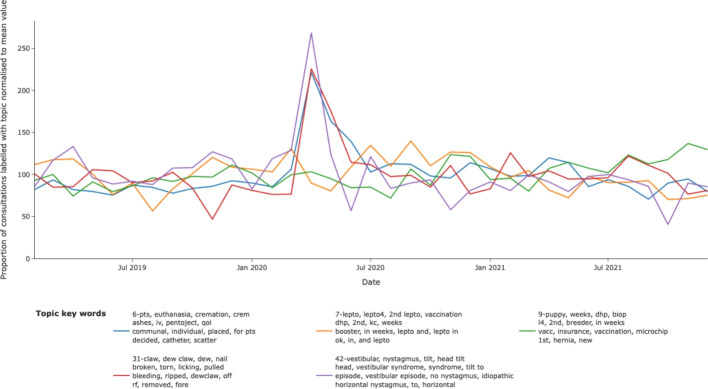
Clinical priorities during COVID-19 lockdown. During lockdown, certain topics became less common and others were over-represented as a proportion of consultations visits. These included visits for painful, distressing or terminal disease. The proportions of routine health care visits did not increase

At the point of the initial lockdown for COVID in April 2020, the number of veterinary consultations decreased significantly [40] however veterinary visits continued and certain topic-labelled narratives formed a higher proportion of consults. These were identifiable as increased description of specific syndromes such as vestibular disease, torn nails, and consultations resulting in euthanasia of the dog. Other topics relating to routine health care did not occur at an increased proportion (Fig.8).

## Discussion

To realise the potential of big data, it is clear that annotation of records for specific features can not be performed manually at the million-record scale due to lack of time, compliance, limitation of the feature set that can be assessed and accuracy of annotations. We have shown that a neural language model method can be used to build a representation of clinical syndromes affecting 1 million canine patients.

As an internal validation of the method, the high proportion of highly-coherent topics in the resultant model, combined with the strong overall average, suggests that the BERTopic model successfully identified distinct and semantically meaningful themes within the corpus, indicating that the model parameters were appropriately tuned and that the resulting topic structure provided a reliable foundation for further thematic analysis and interpretation.

The contrast between high topic coherence and low individual assignment probabilities (median: 0.049) reflects the embedding-based similarity approach and the overlapping nature of clinical presentations, where document embeddings may be positioned between multiple topic clusters in the semantic space. Despite this uncertainty in similarity scores, the high topic coherence confirms that the identified topics represent clinically meaningful and semantically distinct phenotypes. This is appropriate for surveillance applications where the goal is pattern detection. Additionally, a number of known breed predispositions (e.g. Samoyed predisposition to diabetes) and for instance predictable age related topics (e.g. euthanasia) provided some external validation of the model.

The underlying text in the narratives was not preprocessed prior to tokenisation and embedding for analysis. Consequently stop words like ‘no’, ‘not’ and ‘if’ do appear as common words for several topics. We elected not to remove these, respecting the advice of the BERTopic package author [13], because the underlying BERT model was not trained with stop-words removed and because BERT models have been demonstrated to capture negation clues in clinical text [20]. Additionally, the resultant topics were coherent and overall meanings for the topics remained, albeit subjectively, easily interpretable, aligning with breed and age and temporal distributions where these were already known.

The topic model presented a rich overview of clinical features in the data, allowing a review of the prevalence of clinical syndromes across breed, age, sex and with time. As an unsupervised method, topic modelling allows the clinical phenotypes inherent to the data to surface without stipulating what those phenotypes should be. Consequently, a rich set of features can be exposed, and a representative sample of which are highlighted here.

For dogs, breed predisposition to disease is always a complex challenge, even when big datasets are available, because the clinical phenotype under study may require manual annotation. The number of records that can be annotated leads to a significant reduction in group sizes when dividing down to individual breeds, of which there are many 100 s in dogs. Thus, compromises are made in studies whereby only limited breed sets can be evaluated. With automated labelling, far more records are annotated, and more representatives from each breed can be evaluated. Additionally, Topic modelling immediately incorporates numerous phenotypes in a single study.

Here, we evaluated records annotated with topics relating to four common endocrinopathies. In the case of diabetes mellitus, breed predispositions noted in previous big-data studies were clearly identifiable for Border terriers, West Highland White Terriers and Yorkshire Terriers [15]. In that study, case numbers were limited due to a methodology requiring keyword search and manual reading. The wider sampling facilitated through topic modelling as described here also allowed the identification of other, less common, breeds with possible disease predispositions, such as Scottish terrier, Siberian Husky, Samoyed [19] and Bichon Friese, additionally highlighting Boxers, Pugs and French bulldogs as under-represented breeds.

Hypothyroidism was more common in Dobermans, Scottish terriers, Boxer and Samoyed in line with a recent review [30]. Hypoadrenocorticism was seen in Beared collies, Parson Russel Terriers, Poodles and Labradoodles and Hyperadrenocorticism in West Highland White terrier, Border terrier, Bichon Friese and Scottish Terriers.

Similar reviews of the phenotype were possible, such as the evaluation of the distribution of topics relating to heart murmur and upper and lower respiratory tract disease. In this case, heart murmurs were discussed more often in Cavalier King Charles Spaniels (a long recognised predisposition [7]) and less often in Pugs, whereas in this group of breeds, sneezing was more common in Pugs and Chihuahuas and less common in French Bulldogs. A holistic study such as shown here allows for a broader and more efficient assignment of disease processes allowing, in particular, screening for underlying genetic mutations in populations of breeds at varied risk, including rarer breeds not assessed in smaller studies.

For each consultation in this dataset, full patient data (breed, age, sex, neutering status and geographical location) was available, and a comprehensive logistic regression analysis would have been possible but outside the scope or scale of this study. Here, the key feature of this study is the range of phenotypes that can be explored. For example, where previous studies have been able to capture information about diabetes mellitus, the approach demonstrated here concurrently captured data about three other endocrinopathies with no extra investment of time. To the author’s knowledge, a comprehensive single study identifying wide ranging disease predispositions in this manner has not been performed.

The same model, when examined using the topics_per_class method using age as a class, allowed a rapid evaluation of the distribution of given topics with age revealing a variety of patterns, some of which were quite predictable (puppy vaccination in young dogs, booster vaccination with slow decline according to age, euthanasia more common in older animals, dental disease steadily increasing with age) with others less intuitive at first glance (lump/mass discussion in middle age, vestibular disease increasing extremely steeply after 10 years of age). This methodology is ready to combine classes of age and breed in order to highlight how individual breeds variably express specific phenotypes with age and can add to discussions of the different rates of ageing in different breeds [18] and complements other ML-based approaches to characterising factors leading to euthanasia in clinical notes [11].

When topics were analysed per class based on patient sex, numerous examples of topics were more common in one sex or the other. Unsurprisingly, consultations relating to male neutering and female neutering and mammary disease were strongly biased, but additionally, key known predispositions were immediately surfaced, including seizures in male dogs [9], grass seed injury in male dogs [3], urinary tract disease in female dogs [5]. Additionally, consultations describing dog aggression were more common in male dogs, in line with previous studies that indicate male dogs are more prone to human-oriented aggression [22]. Critically, a number of female dog narratives were labelled with the topic with keywords including testicles and castration. These frequently related to descriptions of undescended testicles in puppies brought in with their dam. In some cases these illustrated rare errors where the original data (from the practice management system of the contributing practice) contained the wrong sex highlighting an opportunity to feed back to our contributors on the accuracy of their clinical notes.

Utilising BERTopic’s topic_over_time method offered the opportunity to evaluate various phenotypic features temporally in three ways, namely seasonal disease, unexpected changes in proportion of labelled narratives (potential outbreaks) and long term trends. Here, we demonstrate that phenotypes with known seasonal variation were immediately identified illustrating known patterns of tick infestation, grass seed foreign bodies [1, 3, 42] and fireworks anxiety peaking around bonfire night. Critically this data can be reviewed over time to evaluate the impact of climate change on timing of these effects and in addition, better understanding of the timing of ectoparasite activity will help to inform more focused use of ectoparasiticides which are known to contaminate local environments [32].

The outbreak of canine gastrointestinal disease seen in 2020 [34] was readily identified with subsequent seasonal peaks which have since been identified [6]. Interestingly, the temporal dataset also identified a substantial change in the proportion of narratives labelled with a topic describing kennel cough-like signs (acute respiratory disease) during 2021. To the authors’ knowledge, this has not been demonstrated in any other reports and warrants further investigation. We demonstrated three examples of long term trends in drug usage (as described in clinical notes) suggesting a decrease in the use of a specific meloxicam-containing product (Metacam®), which may reflect the licensing of other products containing the same drug alongside the emergence of a number of COX-2 selective antagonists in recent years. A steady increase in described use of oclacitinib (Apoquel®, a drug used to manage allergic skin disease) was also seen, and a dramatic increase in the description of the use of bedinvetmab (Librella®, a drug used to manage arthritis), which coincided with its release in 2021.

This temporal approach also allowed for the evaluation of the impact of COVID-19 lock-downs. Thus, syndromes associated with pain (torn nails), distress (vestibular disease) and terminal illness and euthanasia represented a larger proportion of visits. These would all be associated with acute distress for the patient and their owners leading owners to pursue immediate care where perhaps vaccination and routine health care were considered acceptable to delay [21].

The sentence-embedding model used has a constrained input length of 512 tokens which means that a fraction (approximately 0.2%) of records will have been truncated prior to embedding and subsequent clustering which may have led to some loss of detail and range of topics detected by the system. In future studies, models with larger input lengths will be usable. The study’s unsupervised topic modelling on a large veterinary clinical dataset offers a broad view of clinical syndromes among one million canine patients, however, the constraints set on cluster size to avoid vacuous or duplicated topics prevent rarer topics from being exposed, which may lead to important but very uncommon syndromes being missed. While topics are often easily interpreted from their key-words, their remains a requirement to audit the underlying narratives where critical conclusions are being drawn. In future work, studies will include setting thresholds for topic probability when attributing a topic to a given consultation in order to improve labelling accuracy. Despite these constraints, the study provides a foundational exploration of clinical phenotypes, offering potential avenues for future investigations with access to full patient histories for in-depth analyses.

## Conclusion

Unsupervised BERTopic topic modelling, when applied to a corpus of veterinary clinical notes, surfaces a diverse array of clinically relevant phenotypes which can be used to expose breed, age and sex predispositions to disease and highlight seasonal and outbreak variations in the occurrence of disease in a single experiment. Additionally, this approach allows the visualisation of trends in the appearance of clinical signs and treatment modalities and changes in treatment priorities during lockdown. This methodology leverages a freely available neural language model but is of particular value in this setting because of the availability of the large SAVSNET dataset with national coverage of clinical records. The model can be used to classify narratives unseen during the initial training, and the full array of topics by breed, age, sex, and date is available to view at https://public.tableau.com/app/profile/savsnet.at.liverpool/viz/topic1000000dogspjtrial/topicrisk

Finally, while this study is focused on population-level phenotypes, the resulting knowledge can directly inform the care of individual patients. Understanding population trends, such as confirmed breed predispositions to endocrinopathies, allows clinicians to perform proactive, evidence-based screening and preventative care. Knowledge of drug use trends (Fig.7) helps anticipate shifts in therapeutic standards, and timely outbreak detection (Fig. 5) allows for immediate, informed treatment of individual animals during high-risk periods. Thus, these large-scale epidemiological insights can translate directly to improved clinical decision-making and patient outcomes.

## Supplementary Information

Supplementary file 1.

## Data Availability

The datasets generated and/or analysed during the current study are not publicly available but are available from the corresponding author on reasonable request subject to ethical approval. A sample of such narratives is available at https://www.liverpool.ac.uk/media/livacuk/savsnet/SAVSNET,sample,vet,data.xlsx.

## References

[1] Arsevska E, Hengl T, Singleton DA, Noble PJM, Caminade C, Eneanya OA, et al. Risk factors for tick attachment in companion animals in Great Britain: a spatiotemporal analysis covering 2014–2021. Parasit Vectors. 2024. 10.1186/s13071-023-06094-4. 10.1186/s13071-023-06094-4 PMC10804489 38254168

[2] Benchimol EI, Smeeth L, Guttmann A, Harron K, Moher D, Petersen I, et al. The reporting of studies conducted using observational routinely-collected health data (record) statement. PLoS Med. 2015;12(10):e1001885. 26440803 10.1371/journal.pmed.1001885 PMC4595218

[3] Brant BJ, Singleton DA, Noble PJM, Radford AD. Seasonality and risk factors for grass seed foreign bodies in dogs. Prev Vet Med. 2021;197:105499. 10.1016/j.prevetmed.2021.105499. 34583207 10.1016/j.prevetmed.2021.105499

[4] Burton J, Farrell S, Noble P-J, Al Moubayed N. Explainable text-tabular models for predicting mortality risk in companion animals. Sci Rep. 2024;14:1–12. 38902282 10.1038/s41598-024-64551-1 PMC11190214

[5] Byron JK. Urinary tract infection. Vet Clin North Am Small Anim Pract. 2019;49:211–21. 10.1016/j.cvsm.2018.11.005. 30591189 10.1016/j.cvsm.2018.11.005

[6] Cunningham-Oakes E, Pilgrim J, Darby AC, Appleton C, Jewell C, Rowlingson B, et al. Emerging variants of canine enteric coronavirus associated with outbreaks of gastroenteric disease - volume 30, number 6–june 2024 - emerging infectious diseases journal - cdc. Emerg Infect Dis. 2024;30:1240–4. 10.3201/EID3006.231184. 38782018 10.3201/eid3006.231184 PMC11139001

[7] Detweiler DK, Patterson DF. The prevalence and types of cardiovascular disease in dogs. Ann N Y Acad Sci. 1965;127(1):481–516. 10.1111/j.1749-6632.1965.tb49421.x. 5217276 10.1111/j.1749-6632.1965.tb49421.x

[8] Devlin J, Chang M-W, Lee K, Toutanova K. Bert: Pre-training of deep bidirectional transformers for language understanding. NAACL HLT 2019 - 2019 Conference of the North American Chapter of the Association for Computational Linguistics: Human Language Technologies - Proceedings of the Conference, 1:4171–4186, (2018). arXiv:1810.04805.

[9] Erlen A, Potschka H, Volk HA, Sauter-Louis C, O’Neill DG. Seizure occurrence in dogs under primary veterinary care in the UK: prevalence and risk factors. J Vet Intern Med. 2018;32(5):1665. 30216557 10.1111/jvim.15290 PMC6189390

[10] Farrell S, Appleton C, Noble PJM, Al Moubayed N. PetBERT: automated ICD-11 syndromic disease coding for outbreak detection in first opinion veterinary electronic health records. Sci Rep. 2023;13(1):1–14. 37865683 10.1038/s41598-023-45155-7 PMC10590382

[11] Farrell S, Anderson K, Noble P-J, Al Moubayed N. Premature mortality analysis of 52,000 deceased cats and dogs exposes socioeconomic disparities. Sci Rep. 2024;14(1):28763. 10.1038/s41598-024-77385-8. 39567516 10.1038/s41598-024-77385-8 PMC11579424

[12] Grootendorst M. Bertopic: Neural topic modeling with a class-based tf-idf procedure. arXiv preprint arXiv:2203.05794, 2022.

[13] Grootendorst M. BERTopic tips & tricks: Removing stop words. https://maartengr.github.io/BERTopic/getting_started/tips_and_tricks/tips_and_tricks.html, 2024. Accessed: 2025-10-27.

[14] Hall PA, Lemoine NR. Comparison of manual data coding errors in two hospitals. J Clin Pathol. 1986;39(6):622–6. 10.1136/JCP.39.6.622. 3722414 10.1136/jcp.39.6.622 PMC499971

[15] Heeley AM, O’Neill DG, Davison LJ, Church DB, Corless EK, Brodbelt DC. Diabetes mellitus in dogs attending UK primary-care practices: frequency, risk factors and survival. Canine Med Genet. 2020;7(1):1–19. 10.1186/S40575-020-00087-7.32835227

[16] Face H. all-minilm-l6-v2 sentence transformers, 2024. URL https://huggingface.co/sentence-transformers/all-MiniLM-L6-v2. [Accessed 2024-06-17].

[17] Ibrahim H, Liu X, Zariffa N, Morris AD, Denniston AK. Reporting guidelines for artificial intelligence in healthcare research. Clin Exp Ophthalmol. 2021;49(5):470–6. 33956386 10.1111/ceo.13943

[18] Jackson J, Radford AD, Belshaw Z, Wallis LJ, Kubinyi E, German AJ, et al. Using veterinary health records at scale to investigate ageing dogs and their common issues in primary care. J Small Anim Pract. 2025;66(2):81–91. 10.1111/jsap.13809. 39663948 10.1111/jsap.13809 PMC11821469

[19] Kimmel SE, Ward CR, Henthorn PS, Hess RS. Familial insulin-dependent diabetes mellitus in Samoyed dogs. J Am Anim Hosp Assoc. 2002;38(3):235–8. 10.5326/0380235. 12022409 10.5326/0380235

[20] Lin C, Bethard S, Dligach D, Sadeque F, Savova G, Miller TA. Does BERT need domain adaptation for clinical negation detection? J Am Med Inform Assoc. 2020;27(4):584–91. 10.1093/jamia/ocaa001. 32044989 10.1093/jamia/ocaa001 PMC7075528

[21] Littlehales R, Noble PJM, Singleton DA, Pinchbeck GL, Radford AD. Impact of Covid-19 on veterinary care. Veterinary Record. 2020;186(19):650–1. 10.1136/VR.M2495. (**ISSN 20427670**). 10.1136/vr.m2495 32587050

[22] Lord M, Loftus BA, Blackwell EJ, Casey RA. Risk factors for human-directed aggression in a referral level clinical population. Vet Rec. 2017;181(2):44–44. 10.1136/VR.103638. 28576767 10.1136/vr.103638

[23] McInnes L, Healy J, Astels S. Hdbscan: hierarchical density based clustering. J Open Source Softw. 2017;2:205. 10.21105/JOSS.00205.

[24] McInnes L, Healy J, Saul N, Großberger L. Umap: uniform manifold approximation and projection. J Open Source Softw. 2018;3:861. 10.21105/JOSS.00861.

[25] Miñarro-Giménez JA, Martínez-Costa C, Karlsson D, Schulz S, Gøeg KR. Qualitative analysis of manual annotations of clinical text with SNOMED CT. PLoS One. 2018. 10.1371/journal.pone.0209547. 10.1371/journal.pone.0209547 PMC6307753 30589855

[26] Nie A, Zehnder A, Page RL, Zhang Y, Pineda AL, Rivas MA, et al. Deeptag inferring diagnoses from veterinary clinical notes. Npj Digital Med. 2018;1:1–8. 10.1038/s41746-018-0067-8. (**ISSN 2398-6352**). 10.1038/s41746-018-0067-8 PMC6550285 31304339

[27] Noble P-J, Appleton C, Radford AD, Nenadic G. Using topic modelling for unsupervised annotation of electronic health records to identify an outbreak of disease in UK dogs. PLoS One. 2021;16(12):e0260402. 10.1371/JOURNAL.PONE.0260402. 34882714 10.1371/journal.pone.0260402 PMC8659617

[28] O’Neill DG, Church DB, McGreevy PD, Thomson PC, Brodbelt DC. Approaches to canine health surveillance. Canine Genet Epidemiol. 2014;1(1):2. 10.1186/2052-6687-1-2. 26401319 10.1186/2052-6687-1-2 PMC4574389

[29] O’Neill DG, Baral L, Church DB, Brodbelt DC, Packer RMA. Demography and disorders of the French Bulldog population under primary veterinary care in the UK in 2013. Canine Genet Epidemiol. 2018;5:1–12. 10.1186/S40575-018-0057-9. 29750111 10.1186/s40575-018-0057-9 PMC5932866

[30] O’neill DG, Su J, Khoo P, Brodbelt DC, Church DB, Pegram C, et al. Frequency, breed predispositions and other demographic risk factors for diagnosis of hypothyroidism in dogs under primary veterinary care in the UK. Canine Med Genetics. 2022;9(1):1–14. 10.1186/S40575-022-00123-8. (**ISSN 2662-9380**). 36217196 10.1186/s40575-022-00123-8 PMC9552398

[31] O’Neill DG, Skipper AM, Barrett K, Church DB, Packer RMA, Brodbelt DC. Demography, common disorders and mortality of Boxer dogs under primary veterinary care in the UK. Canine Med Genet. 2023;10(1):6. 10.1186/S40575-023-00129-W. 37259166 10.1186/s40575-023-00129-w PMC10234096

[32] Perkins R, Goulson D. To flea or not to flea: survey of UK companion animal ectoparasiticide usage and activities affecting pathways to the environment. PeerJ. 2023. 10.7717/PEERJ.15561. 10.7717/peerj.15561 PMC10405796 37554336

[33] Qaiser S, Utara U, Sintok M, Kedah M, Ramsha A, Analytics T. Text mining: use of tf-idf to examine the relevance of words to documents. Int J Comput Appl. 2018;181:25–9. 10.5120/IJCA2018917395.

[34] Radford AD, Singleton DA, Jewell C, Appleton C, Rowlingson B, Hale AC, et al. Outbreak of severe vomiting in dogs associated with a canine enteric coronavirus. United Kingdom. Emerg Infect Dis. 2021;27(2):517–28. 10.3201/EID2702.202452. 33496240 10.3201/eid2702.202452 PMC7853541

[35] Řehůřek R, Sojka P. Software framework for topic modelling with large corpora. In Proceedings of the LREC 2010 Workshop on New Challenges for NLP Frameworks, pages 45–50, Valletta, Malta, 2010. ELRA.

[36] Ribeiro MT, Singh S, Guestrin C. "why should i trust you?" explaining the predictions of any classifier. In Proceedings of the ACM SIGKDD International Conference on Knowledge Discovery and Data Mining, volume 13-17-Augu, pages 1135–1144. Association for Computing Machinery, 2016. ISBN 9781450342322. 10.1145/2939672.2939778.

[37] Rijcken E, Kaymak U, Scheepers F, Mosteiro P, Zervanou K, Spruit M. Topic modeling for interpretable text classification from EHRs. Front Big Data. 2022. 10.3389/FDATA.2022.846930. 10.3389/fdata.2022.846930 PMC9114871 35600326

[38] Sánchez-Vizcaíno F, Noble PJM, Jones PH, Menacere T, Buchan I, Reynolds S, et al. Demographics of dogs, cats, and rabbits attending veterinary practices in Great Britain as recorded in their electronic health records. BMC Vet Res. 2017;13(1):218. 10.1186/s12917-017-1138-9. 28693574 10.1186/s12917-017-1138-9 PMC5504643

[39] Sievert C. Interactive Web-Based Data Visualization with R, plotly, and shiny. Chapman and Hall/CRC, 2020. ISBN 9781138331457. URL https://plotly-r.com.

[40] Singleton DA, Noble PJ, Brant B, Pinchbeck GL, Radford AD. Social distancing impact on companion animal practice. Vet Rec. 2020;186(18):607–8. 10.1136/VR.M2271. 10.1136/vr.m2271 PMC7365568 32527897

[41] Sun S, Zack T, Williams CYK, Sushil M, Butte AJ. Topic modeling on clinical social work notes for exploring social determinants of health factors. JAMIA Open. 2024. 10.1093/JAMIAOPEN/OOAD112. 10.1093/jamiaopen/ooad112 PMC10788143 38223407

[42] Tulloch JSP, McGinley L, Sánchez-Vizcaíno F, Medlock JM, Radford AD. The passive surveillance of ticks using companion animal electronic health records. Epidemiol Infect. 2017;145(10):2020–9. 10.1017/S0950268817000826. 28462753 10.1017/S0950268817000826 PMC5968307

[43] Vandenbroucke JP, Von Elm E, Altman DG, Gøtzsche PC, Mulrow CD, Pocock SJ, et al. Strengthening the reporting of observational studies in epidemiology (STROBE): explanation and elaboration. PLoS Med. 2007;4(10):e297. 17941715 10.1371/journal.pmed.0040297 PMC2020496

[44] Varney D, O’Neill D, O’Neill M, Church D, Stell A, Beck S, Smalley MJ, Brodbelt D. Epidemiology of mammary tumours in bitches under veterinary care in the UK in 2016. Veterinary Record, 2023;e3054. ISSN 2042-7670. 10.1002/VETR.3054. 10.1002/vetr.3054 37231594

