# Supplementary material for: Comprehensive representation of health-related phenotypes in one million dogs using topic modelling of electronic health records
Source: J Big Data. 2026 Feb 24;13(1):50. doi: 10.1186/s40537-026-01365-0 (PMC13035608; doi:10.1186/s40537-026-01365-0)
Supplement: Supplementary file 1 [file 40537_2026_1365_MOESM1_ESM.pdf]

Appendix A Model parameters

parameter	value
min_cluster_size	50
metric	‘euclidean’
cluster_selection_method	‘eom’
prediction_data	True
core_dist_n_jobs	8

Table A1 HDBScan parameters

parameter	value
n_gram_range	(1,2)
embedding_model	‘all-MiniLM-L6-v2’
language	‘english” ’
top_n_words	15
min_topic_size	200
hdbscan_model	hdbscan_model
nr_topics	200
low_memory	True
verbose	True
calculate_probabilities	True

Table A2 Parameters sent to BERTopic

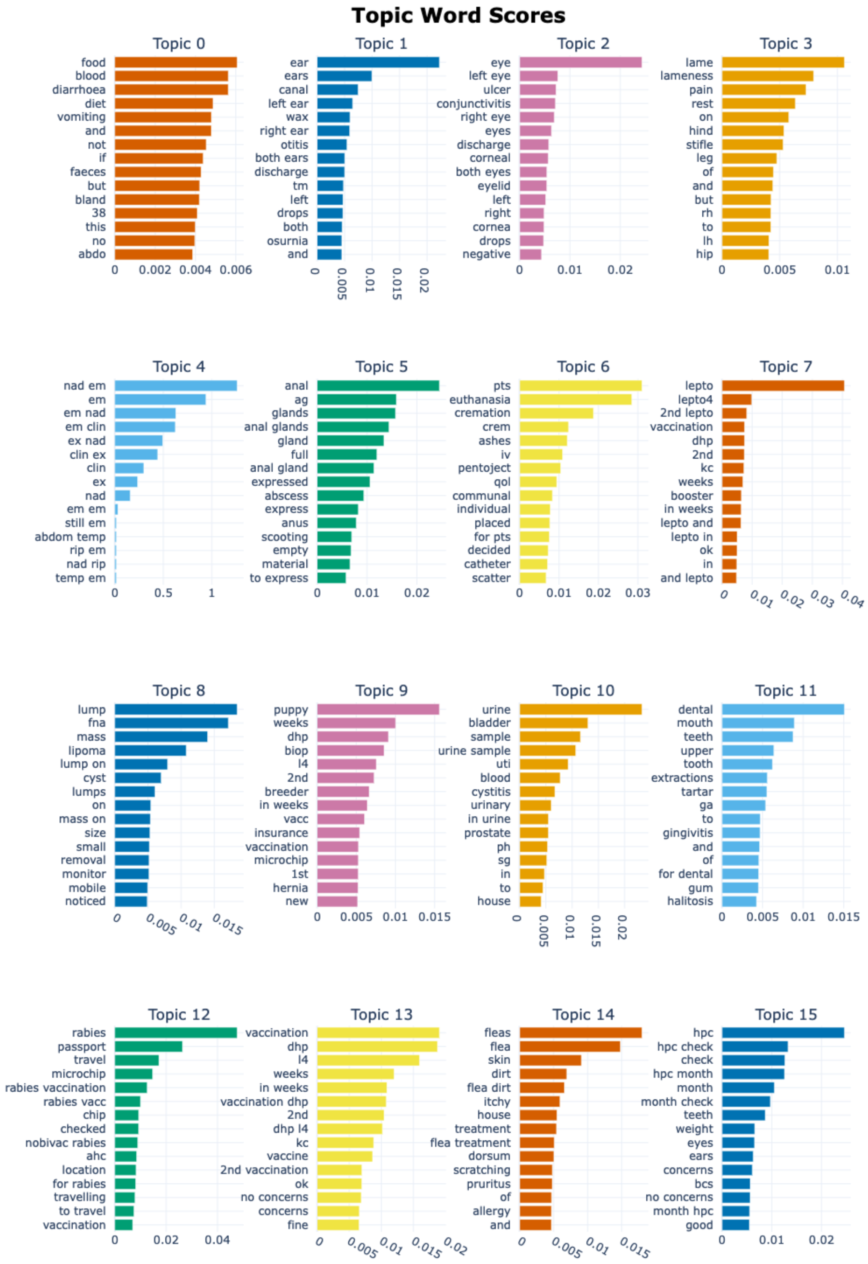

Fig. A1 Topic representations inferred from clinical records, each plot shows the topic number and the 15 most important words or word-pairs contributing to that topic along with the probability weighting for each word in the topic. Word patterns are often explanatory e.g. 'fleas', 'flea', 'skin', 'dirt', 'itchy' reflecting skin disease in presence of fleas or 'ear', 'canal', 'wax', 'otitis' reflecting ear disease.
